# Actuarial Analysis of Survival among Breast Cancer Patients in Lithuania

**DOI:** 10.3390/healthcare9040383

**Published:** 2021-04-01

**Authors:** Aldona Skučaitė, Alma Puvačiauskienė, Rokas Puišys, Jonas Šiaulys

**Affiliations:** 1 Institute of Mathematics, Vilnius University, Naugarduko 24, LT-03225 Vilnius, Lithuania; aldona.skucaite@mif.vu.lt (A.S.); rokas.puisys@mif.stud.vu.lt (R.P.); 2 Ergo Life Insurance SE, Geležinio Vilko 6A, LT-03150 Vilnius, Lithuania; alma.puvaciauskiene@gmail.com

**Keywords:** breast cancer, central death rate, exposed to risk, Kaplan–Meier estimate, survival analysis, cancer awareness campaign, 91G05, 62P05

## Abstract

Breast cancer is the most common cause of mortality due to cancer for women both in Lithuania and worldwide. Chances of survival after diagnosis differ significantly depending on the stage of disease at the time of diagnosis. Extended term periods are required to estimate survival of, e.g., 15–20 years. Moreover, since mortality of the average population changes with time, estimates of survival of cancer patients derived after a long period of observation can become outdated and can be no longer used to estimate survival of patients who were diagnosed later. Therefore, it can be useful to construct analytic functions that describe survival probabilities. Shorter periods of observation can be enough for such construction. We used the data collected by the *Lithuanian Cancer Registry* for our analysis. We estimated the chances of survival for up to 5 years after patients were diagnosed with breast cancer in Lithuania. Then we found analytic survival functions which best fit the observed data. At the end of this paper, we provided some examples for applications and directions for further research. We used mainly the Kaplan–Meier method for our study.

## 1. Introduction

Breast cancer is the most common cause of mortality due to cancer for women both in Lithuania and worldwide in the recent years. Death due to cancer is the second cause of women’s mortality, the first being cardiovascular disease. Analysis of statistical data shows that deaths due to cancer among women of all ages in Lithuania during 2018 amounted to 18% of all deaths, and it was the second cause of mortality after deaths due to cardiovascular disease (63%). Though there were some deaths due to cancer or cardiovascular diseases among girls and women of young age (10–34), mortality rates due to both causes increased quite significantly with age. Both reasons, however, result in a slightly different mortality pattern. Approximately one out of three deaths among women aged 35–74 is due to some type of cancer. Cardiovascular disease is more threatening for women aged 60 and above when mortality rates due to this reason increase from 30% in the age group of 60–64 to almost 80% in the oldest age group (85+). Reduction of mortality due to cancer can result in a lower number of fatalities among women of active age when they are near or at the peak of their career, and/or when their children are young. Breast cancer is usually the most common cause of death among all deaths due to cancer among women. For example, in 2012, deaths due to breast cancer in Lithuania amounted to 24% in the youngest age group (30–54), 18% in the age group 55–74, and 11% among the oldest (aged 75+) [1].

A diagnosis of cancer is still understood by laypeople as a terminal life threatening illness. However, due to advances in medicine, survival after diagnosis can be quite high especially if the disease is found at the onset. Therefore, it is important to estimate survival rates among cancer patients, and to determine whether there are significant differences among patients diagnosed at different stages. Results obtained can then be used for many reasons. For example: Pyenson et al. [2] estimated how screening for lung cancer offered as part of health insurance benefits could help to save lives at a relatively low cost. Estimation of survival can help insurance companies design new health related products, as in some cases the benefit paid depends on the survival of the patient until some time in the future after diagnosis. Results of research can also be useful for public policy makers since the financial burden of cancer can be significant for households. Dieguez et al. [3] analyzed additional costs incurred by breast, lung, and colorectal cancer patients. After survival rates are known, it is easier to assess the financial impact of cancer awareness campaigns, and estimate the amounts of public programs needed to financially support patients and their families.

There is quite a large number of research on the estimation of survival after breast cancer diagnosis. The Kaplan–Meier estimate is one of the most popular tools for such analysis. Some other statistical methods are also used. Fisher et al. [4] analyzed survival among breast cancer patients based on treatment received. Giordano et al. [5] analyzed possible improvements in survival after diagnosis of breast cancer. In Narod et al. [6] it is established whether the mortality rate is influenced by age at the diagnosis, ethnicity and initial treatment received. Narod et al. [7] used the Kaplan–Meier technique, and time to death histograms to estimate mortality of women who died of breast cancer during the 20 year period after diagnosis. In Chen et al. [8] and Giannakeas and Narod [9], the selected groups of patients diagnosed with breast cancer are examined. Seung et al. [10] and Tyurimina et al. [11] drew attention to different scenarios of breast cancer development depending on the subtypes of the tumor. In Smith [12], the breast cancer surveillance guidelines are discussed. We observe that the Kaplan–Meier method is universal and can be applied not only to the study of breast cancer treatment but also to other cancers to compare the survival of different groups of patients after diagnosis, see for instance [13,14,15,16,17,18,19,20,21,22,23,24,25].

Long-term observation of patients is usually required to construct survival functions for even 10–15 years after onset. So, construction of reliable survival models takes a significant amount of time. Moreover, mortality of patients observed for such long periods may be influenced by a number of other factors, such as general changes in mortality in the country. Hence, even after estimating survival for longer periods, it will be unclear whether results may be used to predict mortality of patients diagnosed during recent years. Therefore, some specific statistical and/or actuarial tools are needed to estimate survival for longer periods, but will be based on data collected during, for example 5–10 years. In [26] linear regression is fitted to model breast cancer mortality rates in various regions throughout the world. We went further in this direction. Firstly, we analyzed mortality of breast cancer patients in Lithuania using two different approaches: ratio of deaths to exposure and the Kaplan–Meier estimator. Secondly, we fit the analytic functions to the obtained estimates. The derived analytic functions depend on the stage at onset and can be used to project survival during longer periods.

The rest of our paper is organized as follows. In Section 2, we present some mathematical preliminaries and notations which we use later. In Section 3, we describe data and methods used for our analysis. The main results of our analysis are presented in Section 4. The possible applications of our research are discussed in the concluding Section 5.

## 2. Some Notations and Mathematical Preliminaries

Let us consider a person who was just diagnosed with cancer. Her future lifetime is a nonnegative random variable which we denote by *T*. We assume that there exists a differentiable survival function

S(t)=P(T>t).

We denote the probability to survive until time t+h for the individual being alive at time *t* by the following standard equality

$${}_{h}p_{t}=P\left(T>t+h\;|\;T>t\right)=\frac{S(t+h)}{S(t)}.$$

Alternatively, probability to die until time t+h being alive at time *t* is

$${}_{h}q_{t}=1-{}_{h}p_{t}=\frac{S(t)-S(t+h)}{S(t)}.$$

The instantaneous rate of mortality, or the *force of mortality* at time *t* is defined by equality

$$\mu_{t}=-\frac{S^{\prime }\left(t\right)}{S(t)}.$$

This function $\mu_{t}$ is also called the *hazard function* for the survival function S(t).

Sometimes it is useful to average behavior of the force of mortality in the interval (t,t+1]. In such a case, the *central death rate* or *central mortality rate* $m_{t}$ is used, which is defined as a weighted average of the force of mortality:

$$m_{t}=\frac{\int_{0}^{1}S\left(t+u\right)\;\mu_{t+u}\;du}{\int_{0}^{1}S\left(t+u\right)\;du}.$$

Finally, we define the measure of risk exposure, or *central exposure to risk* $E_{t}$ as the total number of years lived by persons under investigation in the time interval (t,t+1]. If $d_{t}$ is the number of deaths during the period (t,t+1], then the following equality holds for the central death rate

$$m_{t}=\frac{d_{t}}{E_{t}}.$$

## 3. Data and Methodology

The data collected by the Lithuanian Cancer Registry [27] were used for our analysis. The Lithuanian Cancer Registry is a nationwide and population-based cancer registry which covers the whole territory of Lithuania. We analyzed only cases when the patient was diagnosed with cancer for the very first time, i.e., there was no evidence that the patient was diagnosed with any type of cancer before. We analyzed cases diagnosed during the period 1995–2012, and studied the individuals since the onset of disease until death or 31 August 2018, if earlier (end of the study). Cases lost in follow up were treated as right censored, e.g., survival time for such persons we considered to be at least as long as the day of their last observation. We adopted the same approach when treating all survivals until the end of the study period, namely, 31 August 2018. Survival time for those cases was known to be as long as the end of the study period. It is important to note that even patients diagnosed at the end of the diagnostic period (the end of 2012) had the chance to survive at least 5 years. We disregarded the cause of death after diagnosis. We assumed that all deaths after diagnosis were due to cancer, or at least the diagnosis had an influence on the probability of death. One might observe, however, that our results can be easily adapted to the situation when death after a specific period of time since diagnosis (1 year+) was no longer regarded as death due to cancer. On the other hand, some types of insurance (additional critical illness) pay at least part of the benefit conditional on the survival of the insured person for some time after diagnosis. Part of the benefits are no longer paid if the insured person died within a specific period regardless of the reason of death. More about critical illness insurance and types of benefits can be found in Chapter 3 in [28], for instance.

Initially we had a set of 22,437 cases. After initial inspection, we decided to remove 435 cases where the day of death coincided with the day of diagnosis, mainly because majority of such cases were situations when the *death certificate* indicated the cause of death as breast cancer (*death certificate* only cases). Such patients were diagnosed earlier before death, but it was impossible to track the survival time from the moment of diagnosis until death. So, the final set of data consisted of 22,002 records (N= 22,002). Since we believe that the stage of cancer determined at the time of diagnosis can significantly influence the survival time of the patient, we divided our data into groups according to the stage of cancer (see Table 1). Finally, we excluded 738 more cases from further analysis, since the stage of disease was unclear. Consequently, for the final analysis we selected 21,264 cases.

The main goal of our research was to construct survival functions for patients diagnosed with breast cancer based on the stage of disease at onset. Long observation of patients is required to construct survival functions for even 10–15 years after onset. Mortality of patients observed for such long periods may be influenced by a number of other factors, such as general changes in mortality in the country. Hence, even after estimating survival for longer periods, it will be unclear whether results may be used to predict mortality of patients diagnosed during recent years. In our research, our goal was to construct an analytic survival function based on a shorter observation period. We limited estimation of survival functions from the raw data to five years after onset to allow patients diagnosed at the end of the diagnosis period (the end of 2012) survive and be observed until the end of the study period. Otherwise, the percentage of the censored data would increase and could distort survival probabilities simply because lives were censored because the study period had finished. Based on the observed data, we attempted to construct analytic survival functions. We used two different approaches for the parameter estimation.

### 3.1. Exposure to Risk and Central Mortality Rate

At first we estimated the central mortality rate $m_{t}$ at time *t* measured in months since onset using the traditional actuarial technique. For this purpose we calculated exposure to risk based on lives at each period since the onset of disease, and for each stage at inception separately. We use the so-called exact exposure to risk. Since the time of onset was used as the beginning of the observation period for each patient, we had no entries at the times other than the initial time t=0. We assumed that in any time period (t,t+1] contribution to exposure is 1 by all *enders*, while those who died or withdrew contributed less than 1. We calculated the exact contribution of each life from the beginning of the period until the time of death or withdrawal. In that case, the central mortality rate at time *t* has the standard expression: $m_{t}=\frac{d_{t}}{E_{t}}$, where: $m_{t}$ is the central mortality rate in the interval (t,t+1], $d_{t}$ is the number of deaths during the time period (t,t+1] and $E_{t}$ is the central exposure to risk. We only note that deaths occurring at the exact time *t* are attributed to the period (t−1,t]. This is in line with the traditional actuarial approach.

Since the estimated values of the central mortality rate are influenced by quite significant random fluctuations, we graduated the calculated rates using the R software package and the Whittaker-Henderson graduation method. The Whittaker-Henderson smoothing has a couple of advantages. Firstly, it is non-parametric. Hence, it has no assumption about functional form of the central mortality rate. Secondly, it allows the balance between fidelity to observed data and smoothness of the fitted curve. Fidelity of the data is measured by sum SS of squares of deviations between observed values and fitted values:

$$SS=\sum_{i=1}^{n}{\left(y_{i}^{\;observed}-y_{i}^{\;fitted}\right)}^{2}.$$

The fitted curve is smoother for smaller values of the squares of third differences, $M_{3}$. Fitted values are then calculated by minimizing so-called balance function $SS+\lambda M_{3},$ where λ is the so-called smoothing parameter, and

$$M_{3}=\sum_{i=4}^{n}{\left(y_{i}^{\;fitted}-3y_{i-1}^{\;fitted}+3y_{i-2}^{\;fitted}-y_{i-3}^{\;fitted}\right)}^{2}.$$

One can find more about the Whittaker-Henderson graduation in Chapter 11 of [29], for instance.

### 3.2. Kaplan–Meier Method

Alternatively, the Kaplan–Meier estimate can be used to estimate survival probabilities. The main advantages of this method are the following:

- It is suitable for data sets with limited number of cases. Otherwise, using this method may become time consuming. For our data we used an Excel spreadsheet and built-in VBA programming language for this reason.
- It is very suitable for medical trials when time since onset of disease is more important than just the age of the patient, so it is difficult to apply standard actuarial procedures for construction of life tables.
- It is non-parametric, so no advance assumptions about analytic form of survival function are required, nor do parameters need to be estimated. Despite being non-parametric, it is still a statistical estimator, so standard error and confidence intervals may be calculated.
- The estimate for death probability ${}_{h}q_{t}$ is obtained. Interval *h* may be as short as one day, e.g., h=1/365. Hence, the Kaplan–Meier method is suitable for estimation of death probabilities during quite a short period without making any assumptions about distribution of deaths within one year. Moreover, interval *h* may differ for different subintervals and is not determined a priori, but is based on data under investigation.

More information on the Kaplan–Meier method can be found in Chapter 8 of [29], for instance. According to the above procedure, we can obtain the estimator of the survival function by using the following form

$$\hat{S}\left(t\right)=\prod_{t_{i}\leq t}\left(1-\frac{d_{t_{i}}}{l_{t_{i}^{-}}}\right),$$

where $d_{t_{i}}$ is the number of deaths that occurred at time $t_{i}$, and $l_{t_{i}^{-}}$ is the number of patients under observation living immediately before time $t_{i}$.

The approximate value of the standard error of the estimator $\hat{S}\left(t\right)$ can be calculated using the Greenwood formula:

$$\sigma \left(\hat{S}\left(t\right)\right)\approx \hat{S}\left(t\right)\sqrt{\sum_{t_{i}\leq t}\frac{d_{t_{i}}}{l_{t_{i}^{-}}\left(l_{t_{i}^{-}}-d_{t_{i}}\right)}}.$$

## 4. Main Results

### 4.1. Exposure to Risk and Central Mortality Rate

**Figure 1:**
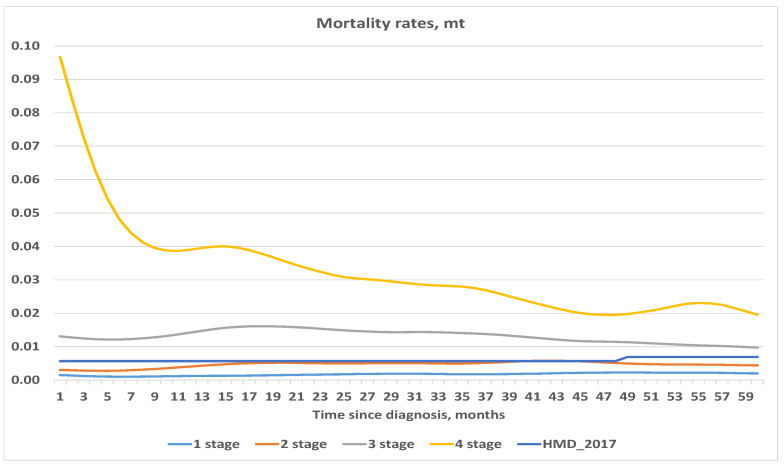
Central mortality rates, stages 1–4.

**Figure 2:**
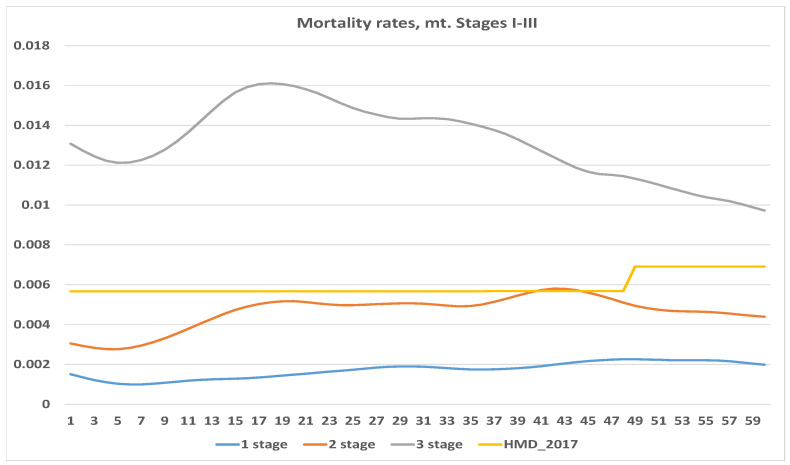
Central mortality rates, stages 1–3.

First we calculated crude estimates of the central death rate. Since the crude estimates show very erratic behavior, we chose a smoothing parameter λ=50. The results obtained are summarized in Figure 1 and Figure 2.

For comparison and illustration purposes, we included the central mortality rates for Lithuanian females aged 57–62 in year 2017 from the *Human Mortality Database* (Available online: www.mortality.org (accessed on 20 April 2020).). The initial age of 57 was chosen because this is the average age of patients diagnosed with Stage 1. Since we measured time in months from onset, and the *Human Mortality Database* provides data for annual periods, we represented the population mortality rate by the almost flat line. As can be expected, the highest mortality rate was observed for Stage 4. Note, however, that the mortality rate for Stage 4 was significantly higher at the beginning, then decreased, and almost converged to the population average. This showed that chances of survival increased over time since diagnosis. A similar pattern (decreasing with time), though not so obvious, is also presented in the curve for Stage 3 (see Figure 2). It is interesting to note that mortality curves for Stages 1 and 2 behave quite extraordinarily. Firstly, they both are below the population average. Secondly, they do not seem to converge to the population average. This can happen for a couple of reasons such as: random fluctuations and the fact that the population average is represented by a mortality rate for females aged 57 at diagnosis. Since the number observed was not so large, it was probably not a good idea to use average (or even median) age for the comparative purposes.

Our estimated mortality rates behaved differently than expected. Usually we expect mortality rates to increase (or at least be constant) with age, except maybe with newborns. Our estimated rates are usually not monotonous, and do not seem to converge to the population average. This means that no one widely used mortality analytic law can be used for projections, and we should use another method for mortality estimation.

### 4.2. Kaplan–Meier Method

Since traditional actuarial techniques seem not to be the best tool for survival estimation, we chose to redo calculations with the Kaplan–Meier estimate. We observed each life from onset of disease until death or withdrawal, and constructed estimator of the survival function. Since the Kaplan–Meier technique is non-parametric, we estimated the mortality of four subsets (based on stage of disease at onset) by stratifying the data into four subgroups (see Table 1), and then we applied the Kaplan–Meier procedure four times.

**Figure 3:**
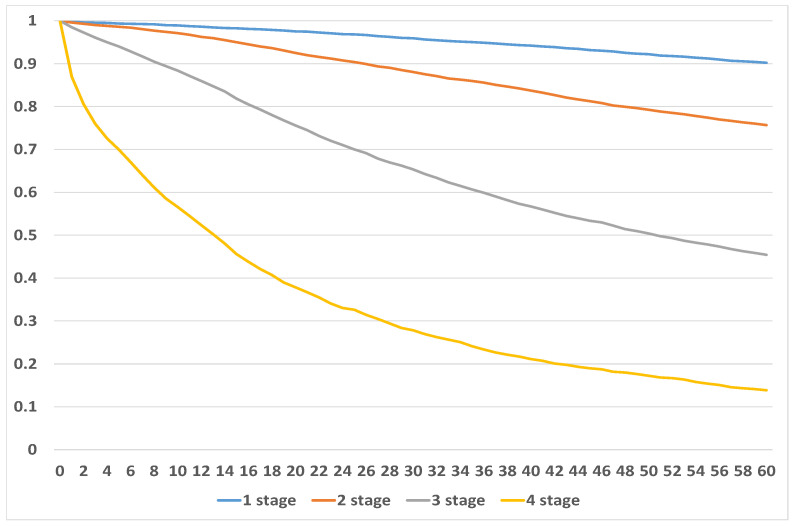
Survival by stage of disease at the moment of diagnosis.

Results of our analysis are summarized in Figure 3 and Table 2.

For simplicity, we assume that every month has 30 days. The analysis showed that there is still a chance to survive at least five years from the onset of disease even if diagnosed with Stage 4. However, as may be reasonably expected, chances of survival decreased quite significantly with the stage found at diagnosis. Those with Stage 1 have slightly more than 90% chance of survival for at least 5 years, while chances for those diagnosed with Stage 4 are only slightly less than 14%. The reader should also observe that even after diagnosis of Stage 1, the chances of survival are lower compared to the female population average, see Table 3), where population mortality tables were obtained from the *Human Mortality Database* (www.mortality.org). The analysis shows that the probability of 5 year survival for patients diagnosed with Stage 1 is 6.5 times higher than for patients diagnosed with Stage 4.

### 4.3. Construction of Analytic Survival Functions

**Figure 4:**
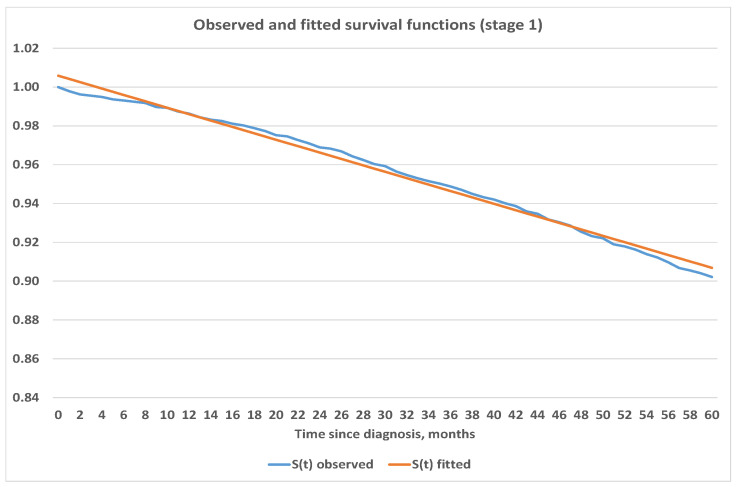
Observed and fitted survival functions based on stage 1 at diagnosis.

**Figure 5:**
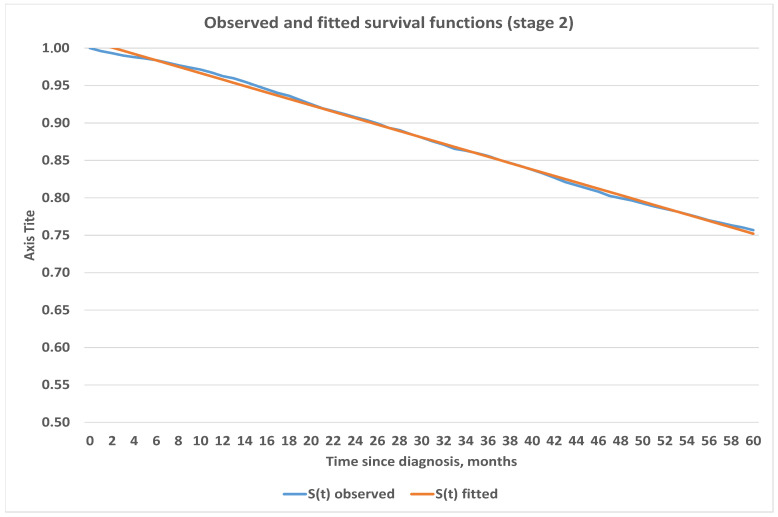
Observed and fitted survival functions based on stage 2 at diagnosis.

**Figure 6:**
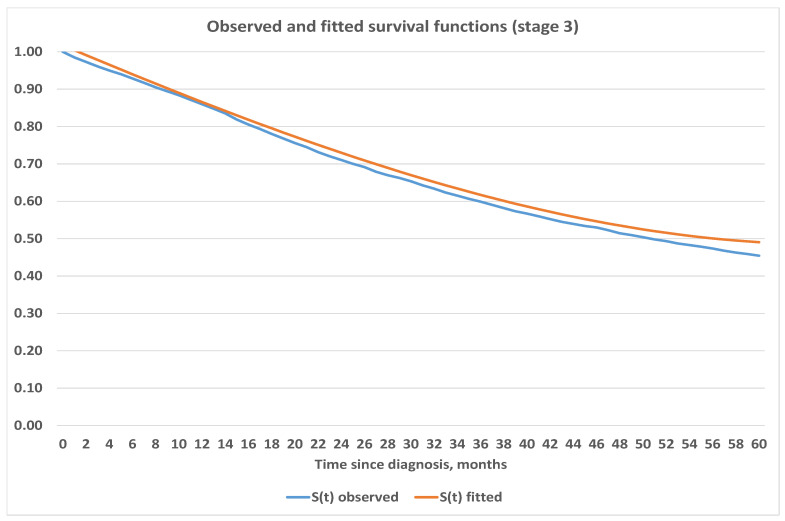
Observed and fitted survival functions based on stage 3 at diagnosis.

**Figure 7:**
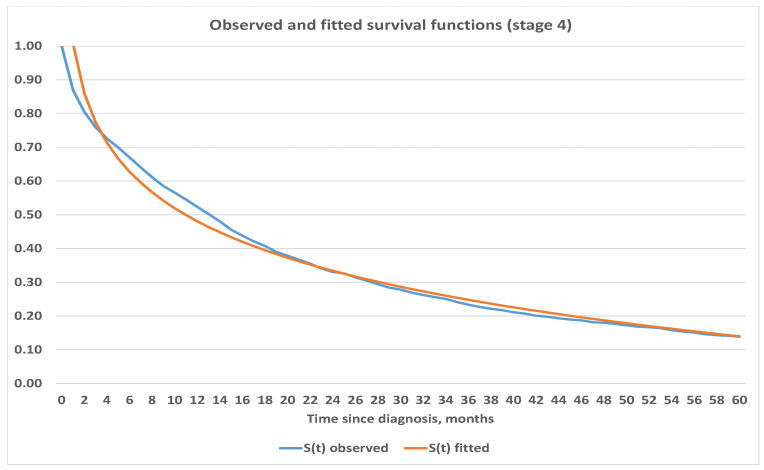
Observed and fitted survival functions based on stage 4 at diagnosis.

We believe that estimates obtained for survival functions using the Kaplan–Meier method are in line with expectations and are consistent. Therefore, we used the obtained survival functions in Figure 3 to project survival into further periods. We used Excel and its built-in capabilities to fit analytic functions which best represent survival depending on the stage at onset. Our analysis shows that survival for Stages 1 and 2 are best fitted by linear function, 3rd degree polynomial was used for Stage 3 and logarithmic function was used for Stage 4. Exact parametric functions with corresponding mean squared errors are presented below in Table 4 while graphical representation can be seen in Figure 4, Figure 5, Figure 6 and Figure 7.

## 5. Applications, Discussion and Conclusions

**Figure 8:**
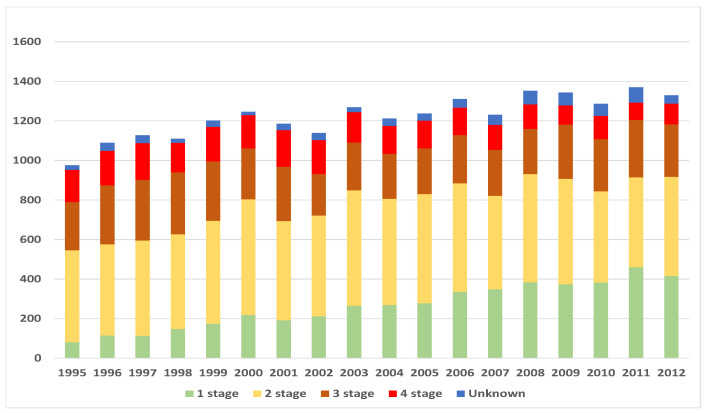
Numbers of new cases by stage and calendar year.

**Figure 9:**
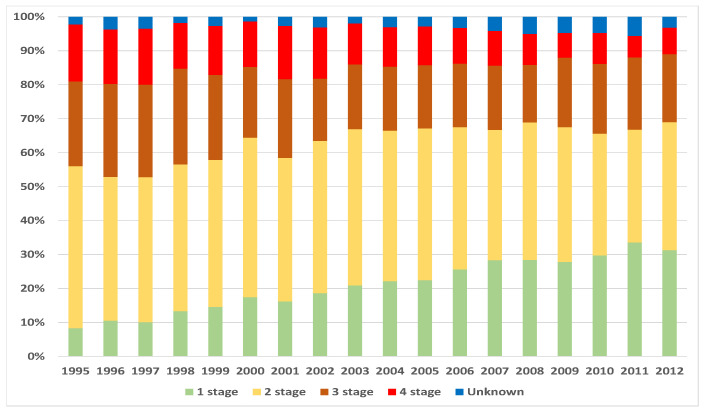
Percentage distribution by stage at diagnosis.

After the construction of estimates of survival functions and their projections, it became clear that steps taken to diagnose breast cancer during Stage 1 of disease are not only psychologically, but also financially sensible. The main measures employed are usually regular medical check-ups, ad hoc check-ups (e.g., for medical underwriting when taking out a life insurance policy) and *Cancer Awareness* campaigns. In Lithuania, currently there are two main breast *Cancer Awareness* programs: the privately financed and managed project *Nedelsk* (Do not delay) and the public program for women aged 50–69. The latter program is financed by the public health care system and covers mammography screening every two years. Both programs started within a two year period: *Nedelsk* in 2002, and the public program in 2004. It was interesting to see whether measures taken have had an effect. For this reason, we analyzed the numbers of diagnosed cases by year and distribution of disease by stage at onset. Results are summarized in Figure 8 and Figure 9. Though there was no significant increase in the number of diagnosed cases, however, the percentage of the mostly threatening Stage 4 decreased from 17% during 1995, to 8% in 2012. At the same time, the percentage of Stage 1 increased from 8% during 1995, to 31% in 2012.

Figures suggest that it is probably worth investing in cancer awareness campaigns. Effectiveness of public cancer awareness campaigns was analyzed more deeply in [30], so interested reader is referred to this source for more information.

Using our projected survival functions and potential changes in stage at onset with and without the *Cancer Awareness* campaign, it is possible to evaluate live years saved or lost due to early or late cancer diagnosis. This may be further used for financial motivation of government spending for public health programs. This issue, however, is beyond the scope of our paper, but may be evaluated in the future.

We believe that further research in this area is worth the investment and should be carried out. One potential direction is to explore whether and—if yes—how quickly the mortality of patients converges to the population average. This may help insurance companies to construct selected mortality tables. We also noticed that the average age of patients increased with the stage at onset. It is interesting whether this is simply a coincidence or some other reason lies behind this fact, e.g., maybe the onset of disease began when patients were on average 57 years old, but simply were not diagnosed for some reason. This may help the government to decide at what age it is more effective to start diagnostic screening programs. Surely, analysis of mortality among breast cancer patients should be repeated after some years to see whether newly obtained results coincide with our results. Analysis done on a regular basis may help to detect whether changes in population mortality (longevity, or pandemic such as COVID-19) have an effect on mortality among breast cancer patients.

## Figures and Tables

**Table 1:** Distribution of cases by stage of disease at diagnosis.

Stage, *i*	Number of Cases, $N_{i}$	Deaths, $D_{i}$	Censored, $C_{i}$ *
1	4767	1258	3509
2	9190	4631	4559
3	4707	3588	1119
4	2600	2480	120
Unknown	738	-	-
Total	22,002	11,957	9307

{*}—including those due to end of study period.

**Table 2:** Survival probabilities and standard errors by stage of disease at the moment of diagnosis.

Duration Since Diagnosis in Months	Stage at Diagnosis							
	1		2		3		4	
	S(t)	Standard Error	S(t)	Standard Error	S(t)	Standard Error	S(t)	Standard Error
0	1	0	1	0	1	0	1	0
1	0.997902	0.000663	0.9961	0.000652	0.984916	0.001777	0.869615	0.006604
2	0.996224	0.000888	0.9931	0.000861	0.972806	0.002371	0.805769	0.007758
3	0.995595	0.000959	0.9901	0.001033	0.960909	0.002825	0.759615	0.00838
4	0.994965	0.001025	0.988	0.001134	0.950074	0.003174	0.725769	0.008749
5	0.993707	0.001145	0.9861	0.001222	0.940089	0.003459	0.699615	0.00899
6	0.993077	0.001201	0.9839	0.001313	0.928617	0.003753	0.670385	0.009219
7	0.992448	0.001254	0.9806	0.001438	0.916932	0.004023	0.640385	0.009411
8	0.991818	0.001305	0.9771	0.001559	0.904823	0.004277	0.611154	0.00956
9	0.98972	0.001461	0.9742	0.001653	0.8942	0.004483	0.585385	0.009662
10	0.989301	0.00149	0.9713	0.001742	0.88379	0.004671	0.565769	0.009721
11	0.987412	0.001615	0.9674	0.001854	0.87168	0.004875	0.545	0.009766
12	0.986363	0.00168	0.9627	0.001977	0.859783	0.005061	0.523462	0.009795
13	0.984475	0.001791	0.9597	0.002051	0.84746	0.005241	0.502308	0.009806
14	0.983216	0.001861	0.9551	0.002161	0.835135	0.005409	0.480385	0.009798
15	0.982586	0.001895	0.9499	0.002275	0.81856	0.005617	0.455769	0.009767
16	0.981117	0.001972	0.945	0.002377	0.805172	0.005773	0.438077	0.00973
17	0.980278	0.002014	0.9401	0.002474	0.793272	0.005903	0.421154	0.009683
18	0.978809	0.002086	0.9363	0.002547	0.780307	0.006035	0.407308	0.009636
19	0.97734	0.002156	0.9308	0.002648	0.767979	0.006153	0.39	0.009566
20	0.975241	0.002251	0.9251	0.002745	0.755863	0.006262	0.378462	0.009512
21	0.974612	0.002279	0.9197	0.002835	0.74481	0.006355	0.366923	0.009452
22	0.972723	0.00236	0.9159	0.002896	0.731419	0.006461	0.355	0.009384
23	0.971044	0.002429	0.912	0.002956	0.720365	0.006543	0.340769	0.009295
24	0.968945	0.002513	0.9077	0.003019	0.710375	0.006612	0.330385	0.009224
25	0.968316	0.002537	0.9039	0.003075	0.700172	0.006679	0.326154	0.009194
26	0.966846	0.002593	0.8992	0.00314	0.691457	0.006733	0.314231	0.009104
27	0.964328	0.002687	0.8936	0.003217	0.678704	0.006807	0.304615	0.009026
28	0.962438	0.002754	0.8904	0.003259	0.669776	0.006856	0.29422	0.008937
29	0.960339	0.002827	0.8852	0.003326	0.662549	0.006893	0.283823	0.008842
30	0.959289	0.002863	0.8806	0.003383	0.653409	0.006937	0.278431	0.008791
31	0.95656	0.002953	0.8755	0.003444	0.642567	0.006986	0.269189	0.008699
32	0.95467	0.003014	0.8709	0.003498	0.633637	0.007024	0.26225	0.008627
33	0.952991	0.003066	0.8655	0.003559	0.623218	0.007064	0.256457	0.008566
34	0.951521	0.003111	0.8629	0.003588	0.614925	0.007094	0.250663	0.008502
35	0.950261	0.00315	0.8596	0.003624	0.606633	0.007122	0.241007	0.008391
36	0.948791	0.003193	0.8558	0.003665	0.598978	0.007145	0.233669	0.008302
37	0.947111	0.003242	0.8508	0.003717	0.59026	0.00717	0.226717	0.008215
38	0.945011	0.003303	0.8465	0.00376	0.581755	0.007191	0.22131	0.008146
39	0.943331	0.00335	0.8422	0.003803	0.57325	0.007211	0.217061	0.008089
40	0.942071	0.003384	0.8374	0.00385	0.567083	0.007224	0.211268	0.008011
41	0.940181	0.003436	0.8325	0.003896	0.559854	0.007237	0.207016	0.007951
42	0.938711	0.003475	0.827	0.003946	0.552199	0.00725	0.200824	0.007863
43	0.935981	0.003546	0.8211	0.003999	0.54497	0.00726	0.197729	0.007817
44	0.934721	0.003579	0.8167	0.004036	0.539442	0.007267	0.193086	0.007748
45	0.931781	0.003653	0.8125	0.004072	0.533701	0.007273	0.189603	0.007694
46	0.930311	0.003689	0.8082	0.004107	0.529873	0.007277	0.187281	0.007658
47	0.928631	0.00373	0.8026	0.004153	0.522431	0.007282	0.181477	0.007566
48	0.92548	0.003805	0.7994	0.004178	0.514348	0.007287	0.179929	0.007541
49	0.923169	0.003859	0.7965	0.004201	0.509456	0.007289	0.176447	0.007484
50	0.922119	0.003883	0.7925	0.00423	0.503925	0.00729	0.172191	0.007413
51	0.918968	0.003954	0.7886	0.00426	0.497755	0.00729	0.168321	0.007346
52	0.917917	0.003977	0.7854	0.004284	0.493283	0.007289	0.166773	0.00732
53	0.916236	0.004014	0.7821	0.004307	0.487103	0.007288	0.163678	0.007265
54	0.913924	0.004064	0.7781	0.004336	0.48262	0.007286	0.157874	0.00716
55	0.912243	0.0041	0.7741	0.004363	0.478561	0.007284	0.154004	0.007089
56	0.909721	0.004153	0.7698	0.004392	0.473636	0.007281	0.150909	0.00703
57	0.906778	0.004213	0.7667	0.004413	0.467633	0.007277	0.145491	0.006925
58	0.905517	0.004238	0.7633	0.004435	0.462698	0.007272	0.14317	0.006879
59	0.904045	0.004268	0.7604	0.004454	0.458834	0.007268	0.141235	0.006841
60	0.902153	0.004305	0.7568	0.004476	0.454323	0.007263	0.138523	0.006786

**Table 3:** Probability to survive 5 years depending on stage at diagnosis.

Stage at Diagnosis	Average Age at Diagnosis	Probability to Survive 5 Years (Population Average), % *	Probability to Survive 5 Years (Patients), %
1	57.55	97.13	90.22
2	58.77	96.85	75.68
3	61.79	95.62	45.43
4	62.97	95.23	13.85

{*} probability to survive is based on integer part of age at diagnosis, so if age is 57.55, we calculate probability for 57 year old women to survive 5 years.

**Table 4:** Analytic survival functions and mean squared errors.

Stage	Estimated Survival Function S(t)	Mean Squared Error
1	−0.00165t+1.005867	$8.42\times 10^{-6}$
2	−0.00429t+1.009426	$3.64\times 10^{-4}$
3	$7\times 10^{-7}\;t^{3}+3\times 10^{-5}\;t^{2}-0.0131\;t+1.0172$	$1.24\times 10^{-5}$
4	−0.21194logt+1.007537	$7.63\times 10^{-4}$

## Data Availability

The data was provided by Lithuanian Cancer Registry https://www.nvi.lt. The data is not publicly available.
